# Hidden peculiar magnetic anisotropy at the interface in a ferromagnetic perovskite-oxide heterostructure

**DOI:** 10.1038/s41598-017-09125-0

**Published:** 2017-08-18

**Authors:** Le Duc Anh, Noboru Okamoto, Munetoshi Seki, Hitoshi Tabata, Masaaki Tanaka, Shinobu Ohya

**Affiliations:** 1 Department of Electrical Engineering and Information Systems, The University of Tokyo, 7-3-1 Hongo, Bunkyo-ku, Tokyo 113-8656 Japan; 2 Institute of Engineering Innovation, Graduate School of Engineering, The University of Tokyo, 7-3-1 Hongo, Bunkyo-ku, Tokyo 113-8656 Japan; 3 Center for Spintronics Research Network (CSRN), The University of Tokyo, 7-3-1 Hongo, Bunkyo-ku, Tokyo 113-8656 Japan

## Abstract

Understanding and controlling the interfacial magnetic properties of ferromagnetic thin films are crucial for spintronic device applications. However, using conventional magnetometry, it is difficult to detect them separately from the bulk properties. Here, by utilizing tunneling anisotropic magnetoresistance in a single-barrier heterostructure composed of La_0.6_Sr_0.4_MnO_3_ (LSMO)/LaAlO_3_ (LAO)/Nb-doped SrTiO_3_ (001), we reveal the presence of a peculiar strong two-fold magnetic anisotropy (MA) along the [110]_c_ direction at the LSMO/LAO interface, which is not observed in bulk LSMO. This MA shows unknown behavior that the easy magnetization axis rotates by 90° at an energy of 0.2 eV below the Fermi level in LSMO. We attribute this phenomenon to the transition between the *e*_g_ and *t*_2g_ bands at the LSMO interface. Our finding and approach to understanding the energy dependence of the MA demonstrate a new possibility of efficient control of the interfacial magnetic properties by controlling the band structures of oxide heterostructures.

## Introduction

Control of magnetic anisotropy (MA) is crucial for low-power magnetization reversal in magnetic thin films. From the perspectives of energy efficiency and scalability, gate-voltage control of the MA via modulation of the carrier density and thus, the Fermi level, is highly desirable^1–4^. For efficient control of MA and for developing materials that are suitable for the MA control, it is necessary to understand the MA of the magnetic thin films over a wide energy range; however, there are few studies from this point of view. In ferromagnetic (FM) materials, the MA energy is related to the magnetization-direction dependence of the density of states (DOS) via the spin orbit interaction^5^. Tunneling anisotropic magnetoresistance (TAMR) is a phenomenon observed in tunnel diodes composed of ferromagnetic (FM) layer/tunnel barrier/nonmagnetic (NM) electrode. TAMR is defined as the change of the tunnel resistance or conductance d*I*/d*V*, which is proportional to the DOS of the electrodes, when rotating the magnetization of the FM layer^5–10^. Thus, TAMR is useful to understand the magnetic-field direction dependence of the DOS. By measuring TAMR at various bias voltages, one can obtain a high-resolution carrier-energy-resolved map of MA of the FM layer^8–10^.

An equally important aspect of TAMR is that it reflects the DOS at the *tunneling interface* of the FM layer, and thus it provides a sensitive probe of the interfacial magnetic properties. Thin film interfaces present both problems and opportunities for exploring new functional devices. As a good example, the “dead layer” at the interface of the perovskite oxide La_1−*x*_Sr_*x*_MnO_3_ (LSMO, the Sr content *x* = 0.3–0.4), which is one of the most promising materials due to its intriguing magnetic and electrical properties such as the colossal magnetoresistance^11, 12^, half-metallic band structure^13, 14^, and high Curie temperature (*T*_C_ ~ 370 K)^11^, is a serious problem for its device applications. For the formation of the dead layer, various possible origins have been proposed, such as intermixing of atoms^15, 16^, oxygen vacancies^17^, lattice distortion^18–20^, and MnO_6_ oxygen octahedral rotations (OOR)^21–23^, which induce orbital, charge, and spin reconstruction at the interfaces of LSMO. These studies on dead layers, however, suggest new ways for controlling the interfacial properties at an atomic level, which are not available in the bulk. To this end, the characterization of the interfacial magnetic properties is highly demanded, but it is difficult with conventional magnetometry because the interfacial properties are usually concealed by the dominant signals from the bulk. Here, by utilizing TAMR in an LSMO/LAO/Nb:STO junction, we obtain the carrier-energy dependence of MA of LSMO for the first time. We also reveal a peculiar strong two-fold symmetry component of MA at the LSMO/LAO interface, which is not observed in bulk LSMO. Moreover, this interfacial MA shows unknown behavior that the symmetry axis of this interface MA rotates by 90° at an energy of 0.2 eV below the Fermi level in LSMO. We attribute this phenomenon to the transition between the *e*_g_ and *t*_2g_ bands at the LSMO interface. Our results suggest that controlling the band structure at interfaces will pave a new way for efficient control of the magnetization of FM thin films, which is essential for devices with low-power consumption.

## Results

### Sample preparation and characterizations

**Figure 1:**
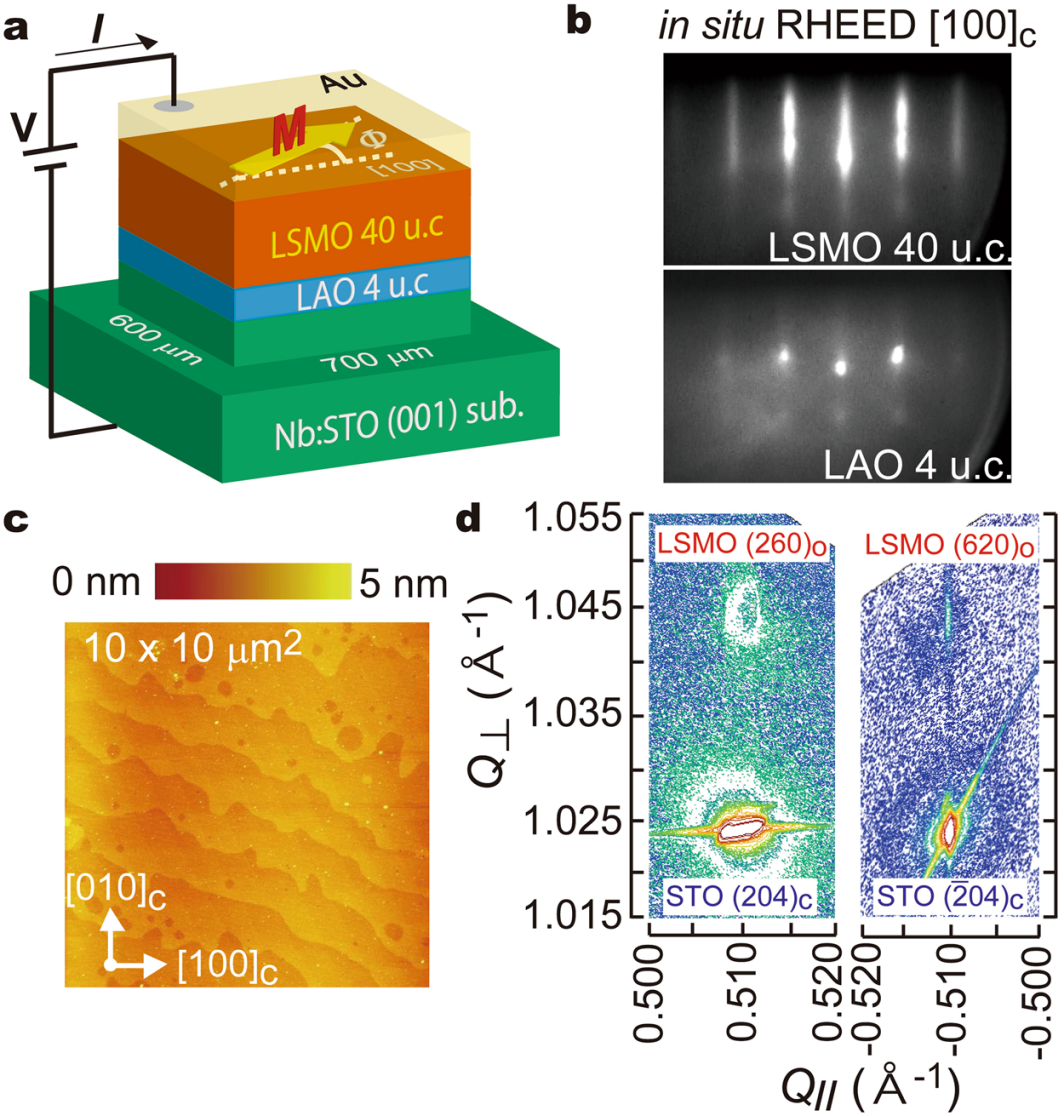
Sample preparation and characterization. (**a**) Device structure and tunneling transport measurement configuration of the LSMO/LAO/Nb:STO tunneling diode structure used in this study. (**b**) *In-situ* reflection high-energy electron-diffraction patterns in the [100]_c_ direction of the LSMO and LAO layers. (**c**) Surface morphology of the LSMO/LAO/Nb:STO sample measured by atomic force microscopy. (**d**) Reciprocal lattice maps of the sample measured at room temperature. Here, *Q*_//_ and *Q*_⊥_ are the components of the reciprocal lattice vector in the in-plane [100]_c_ and out-of-plane [001]_c_ directions, respectively.

The heterostructure used in this study consists of LSMO (40 unit cell (u.c.) = 15.6 nm)/LaAlO_3_ (LAO, 4 u.c. = 1.6 nm) grown on a TiO_2_-terminated Nb-doped SrTiO_3_ (001) substrate (Nb:STO, Nb 0.5% wt.) by molecular beam epitaxy (MBE) (see Fig. 1a and Methods)^24, 25^. The *in-situ* reflection high-energy electron diffraction (RHEED) patterns in the [100] direction of the 4-u.c. LAO and 40-u.c. LSMO layers show streaky patterns, and especially LSMO exhibits a bright pattern (Fig. 1b), indicating that the sample surface is atomically flat. In fact, the atomic force microscopy measurements show flat terraces and atomic steps with a height of ~0.4 nm, which is equal to one pseudocubic u.c. (Fig. 1c). In the x-ray reciprocal lattice map of the sample measured around the (204)_c_ and ${(\bar{2}04)}_{{\rm{c}}}$ reflections of the Nb:STO substrate at room temperature, we see two weaker peaks corresponding to the (260)_o_ and (620)_o_ reflections of the LSMO epilayer (we use the subscripts c and o for the pseudocubic and the orthorhombic crystal structures, respectively) (Fig. 1d). These results confirm that the LSMO layer is coherently grown with respect to the Nb:STO substrate. The (260)_o_ and (620)_o_ peaks of LSMO have nearly the same out-of-plane reciprocal lattice vector *Q*_⊥_, indicating that the (260)_o_ and (620)_o_ atomic plane spacings are equal. This is consistent with the common reports on LSMO thin films grown under tensile strain, indicating that the strain effect in LSMO is accommodated equally between the [100]_c_ and [010]_c_ directions^22^.

For tunneling transport measurements, 600 × 700 μm^2^ mesas were formed by standard photolithography and Ar ion milling. The bias polarity is defined so that the current flows from the LSMO layer to the Nb:STO substrate in the positive bias.

### Magnetic anisotropy components of the LSMO/LAO interface

**Figure 2:**
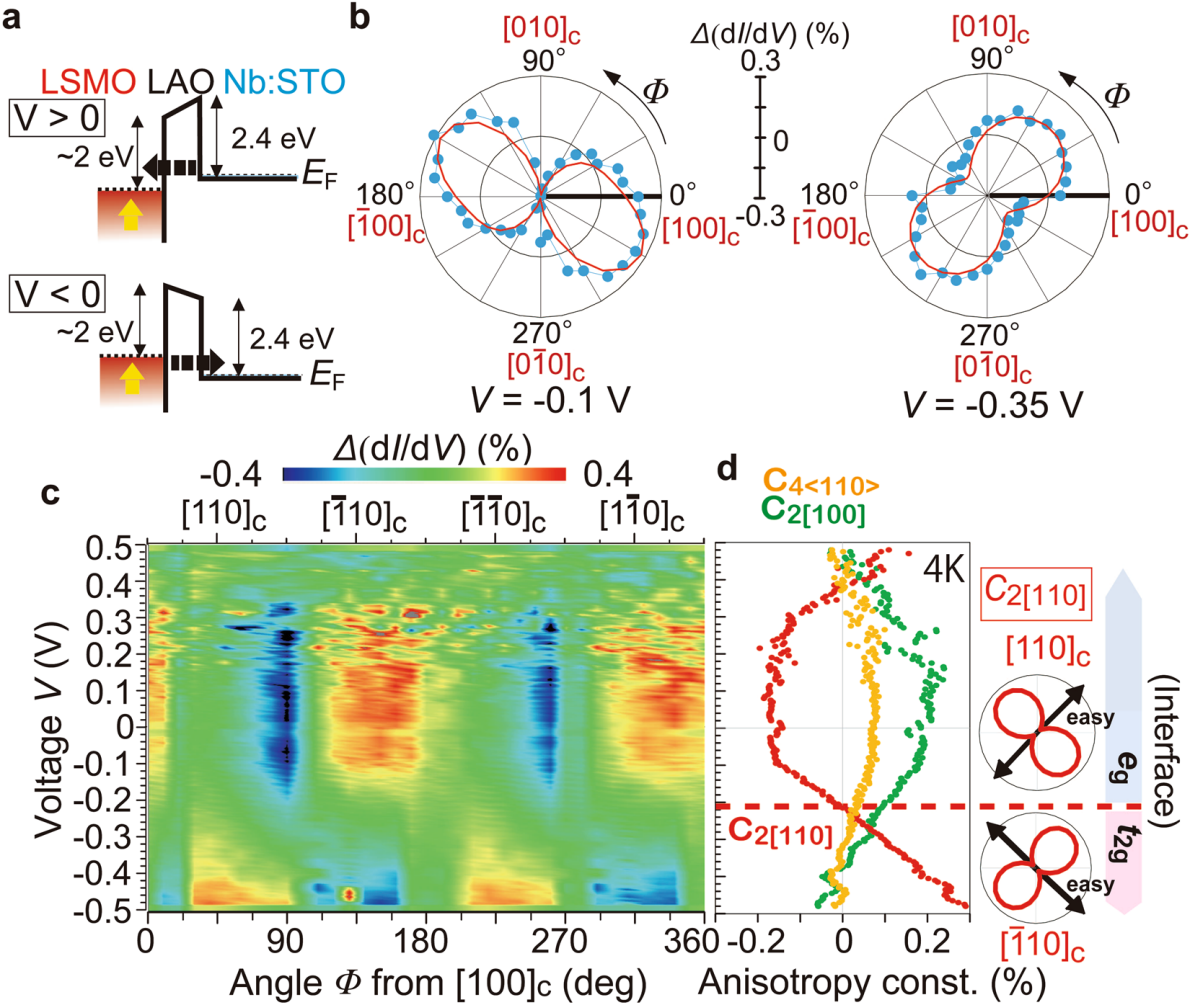
Tunneling anisotropic magnetoresistance results. (**a**) Conduction band (CB) profiles of the LSMO/LAO/Nb:STO tunneling diode under positive and negative bias voltages *V*. The solid and dotted lines represent the top of the CB and the Fermi level *E*_F_. At positive (negative) *V*, the electrons tunnel from Nb:STO to LSMO (from LSMO to Nb:STO). (**b**) Polar plots of $\Delta (\frac{{\rm{d}}I}{{\rm{d}}V})=(\frac{{\rm{d}}I}{{\rm{d}}V}-{\langle \frac{{\rm{d}}I}{{\rm{d}}V}\rangle }_{\Phi })/{\langle \frac{{\rm{d}}I}{{\rm{d}}V}\rangle }_{\Phi }\times 100$ (%) as a function of *Φ* at *V* = −0.1 and −0.35 V (blue points). Here, *Φ* is the magnetic-field angle from the [100]_c_ axis in the counter-clockwise direction in the film plane, and ${\langle \frac{{\rm{d}}I}{{\rm{d}}V}\rangle }_{\Phi }$ is defined as averaged $\frac{dI}{dV}$ over *Φ* at each *V*. The red curves are fitting curves. (**c**) Color plots of ${\Delta }(\frac{{\rm{d}}I}{{\rm{d}}V})$ as a function of *Φ* and *V*. (**d**) *V*-dependence of the symmetry components *C*_4[110]_, *C*_2[100]_, and *C*_2[110]_. The sign of *C*_2[110]_ component changes at *V* = −0.2 V, which corresponds to a 90° rotation of the easy magnetization axis of this component. This is attributed to the transition between the *e*_g_ band and the *t*_2g_ band at the LSMO interface. In the right panel we illustrate the schematic ${\Delta }(\frac{{\rm{d}}I}{{\rm{d}}V})$ − *Φ* data and the easy magnetization axis of the *C*_2[110]_ component when *V* > −0.2 V (top) and *V* < −0.2 V (bottom). All the data were measured at 4 K.

Figure 2a shows the conduction band (CB) profiles of the LSMO/LAO/Nb:STO tunnel diode under positive and negative bias voltages *V*. The Fermi level *E*_F_ is located at 10–20 meV above the CB bottom of STO due to the Nb doping (0.5% wt., the electron density *n* = 1 × 10^20^ cm^−3^)^26^, while *E*_F_ lies in the CB formed by the Mn 3*d*-*e*_g_ states in LSMO. The LAO layer serves as a tunnel barrier with a height of ~2.4 eV for STO and ~2 eV for LSMO^27^. TAMR measurements were conducted as follows: d*I/*d*V* − *V* curves were measured at 4 K while applying a strong external magnetic field of 1 T, which aligned the magnetization direction parallel to the magnetic field, in various in-plane directions with an angle step of 10°. The change in d*I*/d*V* when rotating the external magnetic field is attributed to the change in the DOS at the LSMO/LAO interface or the LAO/STO interface. As illustrated in Fig. 2a, at positive (negative) *V*, electrons tunnel from Nb:STO to LSMO (from LSMO to Nb:STO), and thus d*I*/d*V* probes the DOS of unoccupied (occupied) states in LSMO. At each *V* and *Φ*, where *Φ* is the angle of the magnetization direction from the [100]_c_ axis in the counter-clockwise direction in the film plane, we define ${\Delta }(\frac{{\rm{d}}I}{{\rm{d}}V})$ as $(\frac{{\rm{d}}I}{{\rm{d}}V}-{\langle \frac{{\rm{d}}I}{{\rm{d}}V}\rangle }_{\Phi })/{\langle \frac{{\rm{d}}I}{{\rm{d}}V}\rangle }_{\Phi }\times 100$(%), and ${\langle \frac{{\rm{d}}I}{{\rm{d}}V}\rangle }_{\Phi }$ is defined as averaged $\frac{{\rm{d}}I}{{\rm{d}}V}$ over *Φ* at *V*. As seen in the polar plots of $\Delta (\frac{{\rm{d}}I}{{\rm{d}}V})$ as a function of *Φ* in Fig. 2b, the magnetization direction dependence of the DOS has a mainly two-fold symmetry, but the symmetry axes are different depending on *V*: At *V* = −0.1 V (left panel), the maximum is located at ~150°, between the [010]_c_ and ${[\bar{1}00]}_{{\rm{c}}}$ axes, while it is at 45° (the [110]_c_ axis) when *V* = −0.35 V (right panel). The whole picture of this behavior for all *V* is represented in Fig. 2c, where *Δ*(d*I*/d*V*) is plotted as a function of *Φ* at *V* ranging from −0.5 to 0.5 V. This plot shows a peculiar behavior that the symmetry axis changes at *V* ~ −0.2 V. We fit the data at each bias *V* using the following equation:

1 $$\Delta (\frac{{\rm{d}}I}{{\rm{d}}V})={C}_{4\langle 110\rangle }\,\cos \,[4(\Phi -\frac{\pi }{4})]+{C}_{2[100]}\,\cos \,2\Phi +{C}_{2[110]}\,\cos \,[2(\Phi -\frac{\pi }{4})].$$

Here *C*_4〈110〉_ is the four-fold component with the symmetry axis of 〈110〉_c_, *C*_2[100]_ and *C*_2[110]_ are the two-fold components with the [100]_c_ and [110]_c_ axes, respectively. As shown in Fig. 2b, the fitting curves (red curves) well reproduce the experimental data (blue points). The three anisotropy components estimated in the whole range of *V* are summarized in Fig. 2d. The two-fold symmetry (*C*_2[100]_ or *C*_2[110]_) is stronger than the four-fold symmetry (*C*_4〈110〉]_) in almost all the bias region. The *C*_4〈110〉_ and *C*_2[100]_ components show a similar *V*-dependence, stretching from −0.45 V to 0.4 V. The sign of *C*_2[110]_ changes at *V* ~ −0.2 V, which indicates an opposite dependence of the DOS on the magnetization direction between the regions of *V* > −0.2 V and *V* < −0.2 V. Because the change in the DOS when changing the magnetization direction, *i*.*e*. *Δ*(d*I*/d*V*), is proportional to the MA energy^5^, the sign change of *C*_2[110]_ indicates a 90*°-*shift of the magnetization direction where the MA energy becomes minimum, and consequently indicates a 90°-shift of the easy magnetization axis of the *C*_2[110]_ component from the [110]_c_ to the ${[\bar{1}10]}_{{\rm{c}}}$ directions at *V* ~ −0.2 V when decreasing *V*. This is the main origin of the symmetry axis rotation observed in Fig. 2b and c.

## Discussions

We discuss the origins of these anisotropy components. The biaxial (four-fold) MA along the 〈110〉_c_ axes and the uniaxial (two-fold) MA along the [100]_c_ axis have been reported for LSMO films grown on STO (001) substrates^23, 28–30^, and thus they are bulk-like properties. The biaxial component originates from the in-plane cubic symmetry of LSMO thin films grown on STO. The uniaxial MA along the [100]_c_ axis has been attributed to various origins such as step edges^28, 29^ or different OOR between the [100]_c_ and [010]_c_ directions^23, 30^. In our study, this uniaxial MA along the [100]_c_ axis may also be partially contributed from the high-mobility two dimensional electron gas possibly formed at the LAO/STO interface, where the magnetoconductance has been reported to possess the same two-fold symmetry^31^. On the other hand, the uniaxial MA component along the [110]_c_ direction has never been reported for LSMO. Because the crystal structure of LSMO grown on a substrate with a cubic symmetry such as STO is equivalent between the [110]_c_ and ${[\bar{1}10]}_{{\rm{c}}}$ directions, the uniaxial MA along the [110]_c_ direction is not a bulk property, and thus must be attributed to the LSMO/LAO interface.

**Figure 3:**
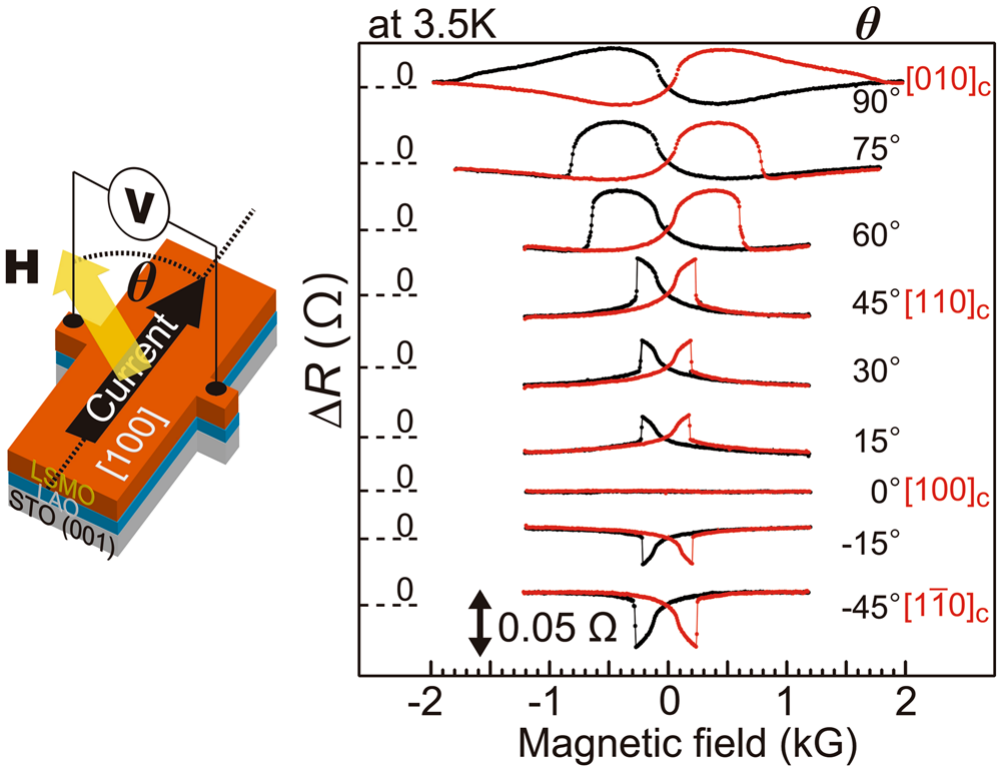
Magnetic anisotropy probed by planar Hall resistance. (Left panel) Schematic illustration of the Hall bar with a size of 50 × 200 μm^2^ formed along the [100]_c_ direction using a reference sample of LSMO (40 u.c.)/LAO (4 u.c.)/non-doped STO (001) substrate. (Right panel) Planar Hall resistance (PHR) Δ*R* of the reference sample with respect to the one at the zero magnetic field measured under various in-plane magnetic field **H** directions. The angle between **H** and the [100]_c_ direction is denoted as *θ*.

To confirm the interface origin of the uniaxial MA component along the [110]_c_ axis, we measured the planar Hall resistance (PHR) of a Hall bar with a size of 50 × 200 μm^2^ formed along the [100]_c_ direction of a reference sample composed of LSMO (40 u.c.)/LAO (4 u.c.). This sample was grown on a non-doped STO (001) substrate under the same conditions as those for the sample used for the TAMR measurements. PHR is proportional to sin *Φ* cos *Φ*, where *Φ* is the angle between the magnetization and the current direction^32^. In Fig. 3, we show the PHR measured for the reference sample at various magnetic field directions, where *θ* is the angle between the magnetic field and the current flown in the [100]_c_ axis. Δ*R* is defined as the PHR with respect to the one at a zero magnetic field. In contrast to the TAMR results, the PHRs of the reference sample measured when the magnetic field is parallel to the [110]_c_ and ${[\bar{1}10]}_{{\rm{c}}}$ directions are identical (*i*.*e*. four-fold like) except the opposite signs, showing *no* clue of the uniaxial MA along the [110]_c_ axis. On the other hand, the PHRs clearly show a dominant uniaxial MA with the easy axis along the [100]_c_ direction. Because PHR mainly reflects the bulk properties of LSMO, our results confirm that the *C*_4〈110〉_ and *C*_2[100]_ components are the MA inherited from bulk LSMO, while the *C*_2[110]_ component is the MA that appears only at the LSMO/LAO interface.

**Figure 4:**
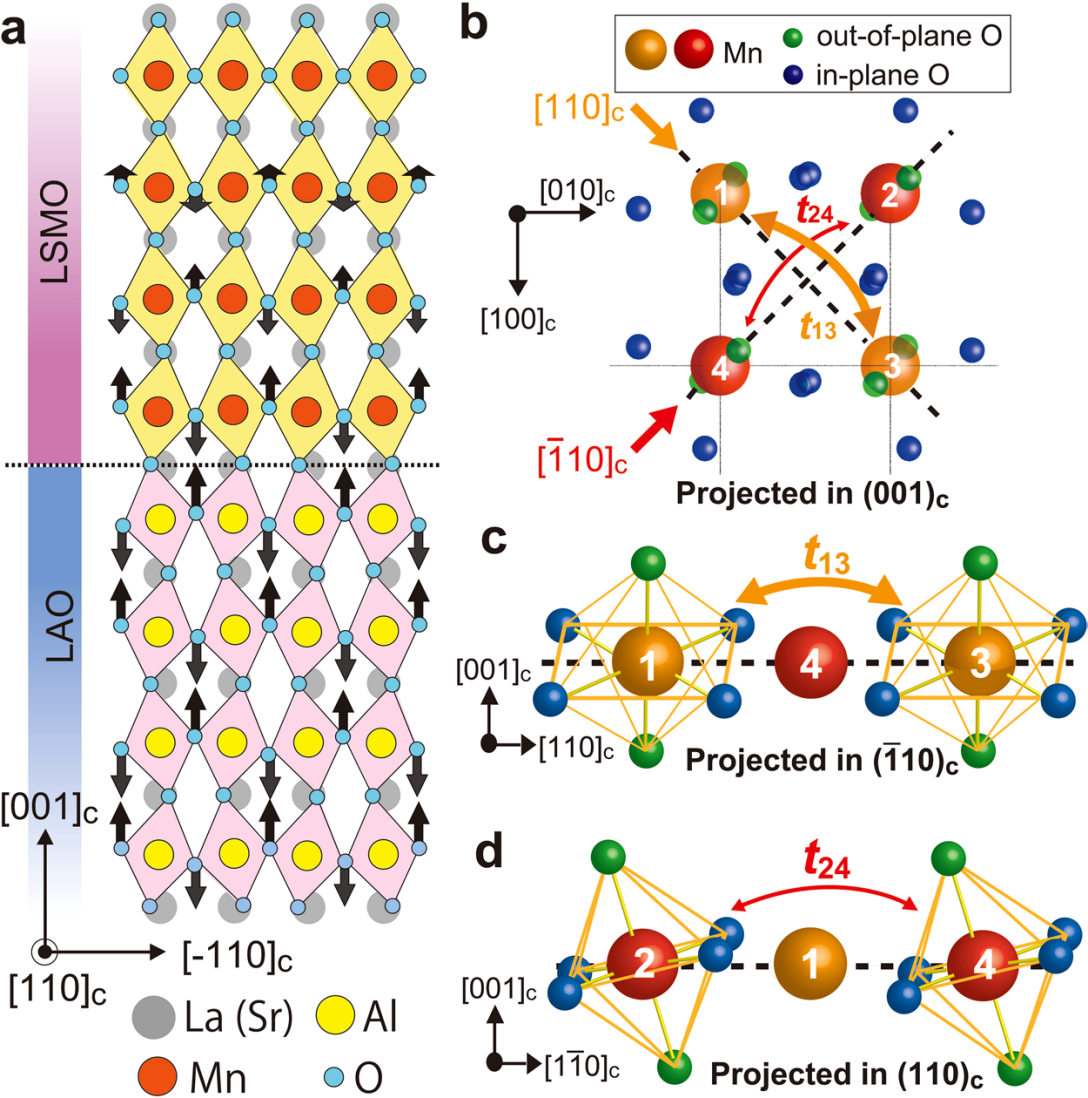
Origin of the two-fold symmetry magnetic anisotropy along the [110]_**c**_ direction. (**a**) Illustration of the crystal structure at the LSMO/LAO interface when looked at from the [110]_c_ direction. In LAO, adjacent corner-sharing oxygen octahedra rotate around the $\,{[11\bar{1}]}_{{\rm{c}}}$ axis in the opposite directions. This lattice distortion is transferred to the first 3–4 u.c. layers of LSMO. (**b–d**) Illustration of four adjacent MnO_6_ octahedra in a (001)_c_ plane of LSMO near the LSMO/LAO interface, when projected in the (001)_c_ (**b**), ${(\bar{1}10)}_{{\rm{c}}}$ (**c**), and (110)_c_ (**d**) planes. Due to the OOR around the $\,{[11\bar{1}]}_{{\rm{c}}}$ axis, the crystal symmetry between the ${[\bar{1}10]}_{{\rm{c}}}$ and [110]_c_ directions is broken. Here, the in-plane and out-of-plane oxygen atoms are drawn in blue and green, respectively. The orange and red spheres represent Mn atoms located along the [110]_c_ and ${[\bar{1}10]}_{{\rm{c}}}$ directions, respectively. The rotation angle is largely exaggerated.

Because the *C*_2[110]_ component is attributed to a symmetry breaking between the [110]_c_ and ${[\bar{1}10]}_{{\rm{c}}}$ directions occurring locally near the LSMO/LAO interface, the OOR mechanism is most likely the origin of *C*_2[110]_. Recently, it has been clarified that adjacent corner-sharing oxygen octahedra in LAO grown on STO (001) rotate in the opposite directions around the $\,{[11\bar{1}]}_{{\rm{c}}}$ axis^33, 34^, and that the OOR in the underlayer is transferred to the first 3–4 u.c. layers of LSMO^21, 22^ (Fig. 4a). Figure 4b shows four adjacent MnO_6_ octahedra in a (001)_c_ plane of LSMO near the LSMO/LAO interface. We see that under the OOR around the $\,{[11\bar{1}]}_{{\rm{c}}}$ axis illustrated in Fig. 4a, the oxygen octahedra located along the [110]_c_ direction rotate in the same direction (see the green oxygen spheres around Mn1 and Mn3). This rotation direction is the opposite to that of the adjacent rows of Mn atoms (see Mn2 and Mn4). When Fig. 4b is projected in the ${(\bar{1}10)}_{{\rm{c}}}$ plane as shown in Fig. 4c, one can see that the vertical O–Mn1–O and O–Mn3–O bonds (see the green spheres) remain nearly perpendicular to the (001)_c_ plane (dotted line). Because the vertical O–Mn1–O bonds are rotated in the same direction as the O–Mn3–O bonds, the hopping integral (*t*_13_) between Mn1 and Mn3 remains nearly unchanged from the value of the bulk. On the other hand, when Fig. 4b is projected in the (110)_c_ plane (Fig. 4d), one can see that the MnO_6_ oxygen octahedra around Mn2 and Mn4 are largely tilted to the left. This decreases the hopping integral (*t*_24_) between Mn2 and Mn4. The difference between *t*_13_ and *t*_24_ yields the anisotropic DOS between the [110]_c_ and ${[\bar{1}10]}_{{\rm{c}}}$ directions and consequently induces *C*_2[110]_ at the LSMO/LAO interface.

The most striking feature found in our study is the sign reversal of *C*_2[110]_ at *V* = −0.2 V (Fig. 2d). This behavior is likely related to the band structure of LSMO, as explained below. In Fig. 2d, one can see that *C*_4〈110〉_ and *C*_2[100]_ show similar *V* dependence in all the *V* region. These results indicate that both originate from the same band located around *E*_F_ of LSMO, *i*.*e*. the up-spin Mn 3*d*-*e*_g_ band. The *C*_4〈110〉_ and *C*_2[100]_ components disappear at ~*V* = −0.45 V, which means that *E*_F_ is located at ~0.45 eV above the bottom of the *e*_g_ band. This is consistent with the results of angle-resolved photoemission spectroscopy (ARPES) measurements for LSMO^35^. Therefore, the emergence of positive *C*_2[110]_ below *V* = −0.2 V is likely associated with the *t*_2g_ band, which is located below the *e*_g_ band. Although the *t*_2g_ state is located at 0.5–1 eV below *E*_F_ in bulk LSMO^35^, it is thought to be largely pushed up by the polar mismatch at the LSMO/LAO interface^36^. Thus, we attribute the sign change of *C*_2[110]_ to the transition from the *e*_g_ band (*V* > −0.2 V) to the *t*_2g_ band (*V* < −0.2 V) at the LSMO interface. As mentioned above, due to the OOR at the LSMO/LAO interface, the DOSs of both the *e*_g_ and *t*_2g_ bands in the [110]_c_ direction are larger than those in the ${[\bar{1}10]}_{{\rm{c}}}$ direction. However, the relationship between the DOS and the magnetization in these two band components is opposite: It is known that the electron transfer via the *e*_g_ orbitals enhances the double exchange interaction and strengthens the ferromagnetism, while the one between the *t*_2g_ orbitals enhances the super-exchange interaction and weakens the ferromagnetism^37^. Therefore, the enhancement of DOS in the [110]_c_ direction relative to that in the ${[\bar{1}10]}_{{\rm{c}}}$ direction makes the [110]_c_ axis the easy magnetization direction in the case of the *e*_g_ orbitals, while hard magnetization direction in the case of the *t*_2g_ orbitals. The transition between these two bands at V = −0.2 V thus leads to the opposite magnetization direction-dependence of the DOS, as consequently observed by TAMR^5^.

In summary, using TAMR measurements, we have successfully obtained a high-resolution map of the MA spectrum of LSMO for the first time. In addition to the biaxial MA along 〈100〉_c_ and the uniaxial MA along [100]_c_, which originate from bulk LSMO, we found a peculiar uniaxial MA along the [110]_c_, which is attributed to the LSMO/LAO interface. The symmetry axis of this interface MA rotates by 90° at an energy of 0.2 eV below *E*_F_ of LSMO, which is attributed to the transition from the *e*_g_ band (>−0.2 eV) to the *t*_2g_ band (<−0.2 eV). These findings hint an efficient way to control the magnetization at the LSMO thin film interfaces, as well as confirm the rich of hidden properties at thin film interfaces that can be revealed only by interface-sensitive probes. This work also suggests the use of the TAMR measurement as a simple but highly sensitive method for characterizing interfacial magnetic properties of magnetic tunnel junctions, which is important for developing spintronic devices.

## Methods

The heterostructure used in this study consists of LSMO (40 unit cell (u.c.) = 15.6 nm)/LaAlO_3_ (LAO, 4 u.c. = 1.6 nm) grown on a TiO_2_-terminated Nb-doped SrTiO_3_ (001) substrate (Nb:STO, Nb 0.5% wt.) by molecular beam epitaxy (MBE) with a shuttered growth technique^24, 25^. The fluxes of La, Sr, Mn, and Al were supplied by Knudsen cells. The LAO and LSMO layers were grown at 730 °C with a background pressure of 2 × 10^−4^ Pa of a mixture of oxygen (80%) and ozone (20%). After the growth, the sample was further annealed at 600 °C in ambient atmosphere for 1 hour to reduce the density of oxygen vacancies.

For tunneling transport measurements, a 50-nm-thick Au film was deposited on top of the sample, and 600 × 700 μm^2^ mesas were then formed by standard photolithography and Ar ion milling. Au wires were bonded to the Au electrode and the backside of the Nb:STO substrate by indium.

The d*I*/d*V*-*V* characteristics were numerically obtained from the *I*-*V* data with a differential interval of 10 mV. See Supplementary Information for more details on how to extract the $\Delta (\frac{dI}{dV})$ data plotted in Fig. 2.

### Data Availability

The datasets of the current study are available from the corresponding author on reasonable request.

## Electronic supplementary material

Supplementary Information
