# Supplementary material for: Hidden peculiar magnetic anisotropy at the interface in a ferromagnetic perovskite-oxide heterostructure
Source: Sci Rep. 2017 Aug 18;7:8715. doi: 10.1038/s41598-017-09125-0 (PMC5562907; doi:10.1038/s41598-017-09125-0)
Supplement: Supplementary file 1 — Supplementary Information [file 41598_2017_9125_MOESM1_ESM.pdf]

Supplementary Information

Hidden peculiar magnetic anisotropy at the interface in a ferromagnetic perovskite-oxide heterostructure

Le Duc Anh,[1,2,*] Noboru Okamoto,[1] Munetoshi Seki,[1] Hitoshi Tabata,[1,3] Masaaki Tanaka,[1,3,**] and Shinobu Ohya[1,2,3,***]

[1]*Department of Electrical Engineering and Information Systems, The University of Tokyo, 7-3-1 Hongo, Bunkyo-ku, Tokyo 113-8656, Japan*
[2]*Institute of Engineering Innovation, Graduate School of Engineering, The University of Tokyo, 7-3-1 Hongo, Bunkyo-ku, Tokyo 113-8656, Japan*
[3]*Center for Spintronics Research Network (CSRN), The University of Tokyo, 7-3-1 Hongo, Bunkyo-ku, Tokyo 113-8656, Japan*

* Corresponding author: anh@cryst.t.u-tokyo.ac.jp
** Corresponding author: masaaki@ee.t.u-tokyo.ac.jp
*** Corresponding author: ohya@cryst.t.u-tokyo.ac.jp

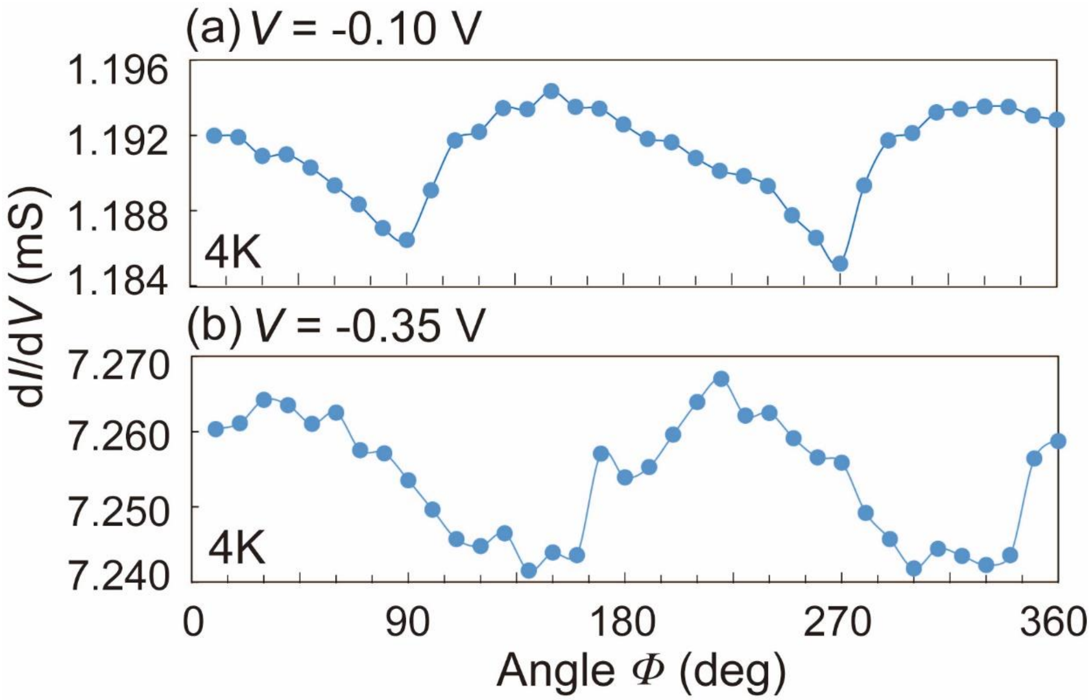

Supplementary Figure S1(a)(b). Raw data of d*I*/d*V* as a function of the angle *Φ* at *V* = -0.10 V (a) and -0.35 V (b), measured at 4 K.

Supplementary Note 1: Procedure to extract the $\Delta\left(\frac{\mathrm{d}I}{\mathrm{d}V}\right)$ data

The procedure how we extract the data plotted in Fig. 2 in the main manuscript is as follows:

Step 1: We measured the *I*-*V* curve at 4 K applying a strong external magnetic field of 1 T in various in-plane directions with an angle step of 10°. We define Φ as the angle of the magnetization direction, which is the same as the magnetic field direction, from the $[100]_c$ axis in the counter-clockwise direction in the film plane. The total data set thus includes 36 *I*-*V* curves measured with different values of Φ.

Step 2: For each Φ, the d*I*/d*V*-*V* curve was numerically obtained from the *I*-*V* curve with a differential interval of 10 mV.

Step 3: For each fixed bias voltage *V*, we calculated $\left\langle\frac{\mathrm{d}I}{\mathrm{d}V}\right\rangle_\Phi$, which is the average value of d*I*/d*V* over all 36 values of Φ.

Step 4: At each fixed *V* and Φ, we calculated the deviation (in percentage) of the d*I*/d*V* value from $\left\langle\frac{\mathrm{d}I}{\mathrm{d}V}\right\rangle_\Phi$ at the same *V*:

$$\Delta\left(\frac{\mathrm{d}I}{\mathrm{d}V}\right) = \left(\frac{\mathrm{d}I}{\mathrm{d}V}-\left\langle\frac{\mathrm{d}I}{\mathrm{d}V}\right\rangle_\Phi\right)/\left\langle\frac{\mathrm{d}I}{\mathrm{d}V}\right\rangle_\Phi \times 100\ (\%). \qquad \text{(S1)}$$

The data plotted in Fig. 2b are the $\Delta\left(\frac{\mathrm{d}I}{\mathrm{d}V}\right)$ values as a function of Φ when *V* is fixed at -0.10 V and -0.35 V. In Supplementary Fig. S1, we plot the raw data of d*I*/d*V* as a function of Φ at *V* = -0.10 V (a) and -0.35 V (b) obtained after Step 2 explained above. At each *V*, d*I*/d*V* shows the same Φ dependence as $\Delta\left(\frac{\mathrm{d}I}{\mathrm{d}V}\right)$.
